# Experience of the Pediatric Department at the Mohammed VI University Hospital Center in Oujda on Trisomy 21 and Congenital Heart Defects: What Is the Reality in the Oriental Region of Morocco?

**DOI:** 10.7759/cureus.86689

**Published:** 2025-06-24

**Authors:** Abdeladim Babakhouya, Chaymae Yechouti, Chaimae Salhi, Aziza Elouali, Maria Rkain

**Affiliations:** 1 Pediatric Department, University Hospital Center of Mohammed VI, Faculty of Medicine and Pharmacy, Mohammed Premier University, Oujda, MAR

**Keywords:** cardiology imaging, congenital cardiac surgery, cyanotic congenital heart disease, down syndrome, trisomy of 21

## Abstract

Introduction

Trisomy 21 (T21), or Down syndrome, is frequently associated with congenital heart defects (CHDs). This study aims to describe the epidemiological, clinical, and para-clinical profile of CHDs in children with trisomy 21 while highlighting the specific challenges encountered in the Oriental region of Morocco.

Methods

This is a retrospective descriptive study conducted over a nine-year period (January 2015 to December 2023) at the Mohammed VI University Hospital Center (CHU Mohammed VI) in Oujda. It included 167 children with trisomy 21 who were referred for cardiologic evaluation, whether symptomatic or screened systematically. All patients underwent echocardiographic assessment performed by a pediatric cardiologist following the guidelines of the American Society of Echocardiography (ASE). Data were collected from medical records and the Hosix software (SIVSA, Vigo, Spain), an electronic health record system.

Results

Among the 167 children, 103 (61.6%) had confirmed congenital heart defects. The average age at diagnosis was 14 months, with the majority being diagnosed after 24 months. Trisomy 21 was confirmed by karyotyping. Most children were referred due to clinical symptoms (feeding difficulties, respiratory distress, cyanosis, sweating, and malaise), while others were referred after an incidental murmur or systematic screening.

The most common defects were complete atrioventricular septal defect (AVSD), followed by ventricular septal defect (VSD), atrial septal defect (ASD), isolated patent ductus arteriosus (PDA), tetralogy of Fallot (ToF), and transposition of the great vessels (TGV), along with rarer anomalies. Pulmonary hypertension (PH), assessed via echocardiographic criteria, was found in 17 patients, with six being deemed inoperable.

Of the 103 children with CHDs, 28 underwent surgical correction, 63 received medical follow-up, and 12 were under palliative care. The low surgical intervention rate was due to various factors: late diagnosis, parental refusal, or limited access to specialized care. Postoperative follow-up was incomplete, limiting outcome evaluation.

Conclusion

This study highlights the challenges of the early diagnosis and optimal management of CHDs in children with trisomy 21 in a resource-limited region. It emphasizes the need to strengthen neonatal screening programs, unify evaluation protocols, and improve access to surgical care to enhance outcomes and long-term survival for these patients.

## Introduction

Congenital anomalies affect approximately one in every 33 newborns globally and represent a significant cause of infant morbidity and mortality. Among these anomalies, trisomy 21 (T21), also known as Down syndrome, is the most common chromosomal disorder, with an estimated incidence ranging from one in 700-1,000 live births. Children with T21 are particularly predisposed to congenital heart defects (CHDs), which are reported in approximately 40%-50% of cases. The most frequently observed lesions include atrioventricular septal defects (AVSDs), ventricular septal defects (VSDs), and atrial septal defects (ASDs). If left untreated, these conditions can result in serious complications such as congestive heart failure and irreversible pulmonary hypertension [1].

In Morocco, national epidemiological data on the prevalence of T21 and its association with CHDs remain limited. A study conducted at the Ibn Sina University Hospital in Rabat reported a high prevalence of CHDs among children with T21, emphasizing their public health significance. However, there is a notable lack of region-specific data, particularly from the Oriental region.

The Oriental region of Morocco faces unique challenges in the diagnosis and management of CHDs in children with T21. These include geographic barriers related to the distance from specialized healthcare centers, economic disparities, and a shortage of trained pediatric cardiologists. These factors often lead to delayed diagnosis and treatment, despite recent improvements in access to prenatal and neonatal echocardiographic screening.

This retrospective study was conducted at the Pediatric Department of the Mohammed VI University Hospital Center (CHU Mohammed VI) in Oujda over a nine-year period. It focuses exclusively on cases of T21 with echocardiographically confirmed CHDs. The primary objective is to describe the epidemiological, clinical, and cardiac features of this population; assess diagnostic delays; and evaluate management strategies within the context of the Oriental region’s healthcare limitations.

The retrospective design allows for the analysis of real-world clinical data drawn from patient records, providing a meaningful overview of local healthcare delivery. In this study, the term “experience” refers to the full spectrum of the clinical journey, including the types of CHDs identified, the timing of diagnosis, the treatments administered, and access to surgical care.

To our knowledge, no similar study has been published in this region. This work aims to contribute new data to the national literature and to support the development of regionally adapted health policies to improve the early detection and management of CHDs in children with T21.

## Materials and methods

Study design and setting

This was a retrospective descriptive study conducted over a nine-year period, from January 2015 to December 2023, in the Pediatric Cardiology Unit of the Pediatric Department at the Mohammed VI University Hospital Center (CHU Mohammed VI) in Oujda, located in the Oriental region of Morocco. This unit serves as the primary referral center for pediatric cardiology across the entire region.

Study population

The study included all children with Down syndrome (trisomy 21) referred for cardiac evaluation during the study period. The patients were referred either during hospitalization in the pediatrics, neonatology, or neonatal intensive care units or through outpatient consultations or transfers from other hospitals or healthcare facilities within the Oriental region.

Out of 167 children with T21 evaluated, 103 were found to have congenital heart defects (CHDs) confirmed by echocardiography and were included in the final analysis. The remaining 64 children without detectable CHDs were noted for contextual reference but excluded from the primary descriptive analysis.

Inclusion and exclusion criteria

Inclusion Criteria

Children aged 0-15 years with a confirmed diagnosis of T21 (either via karyotype or, when unavailable, based on clinical assessment by a pediatrician) and who had echocardiographic confirmation of a CHD were included in the study.

Exclusion Criteria

Patients with incomplete medical records, defined as the absence of a confirmed T21 diagnosis, missing echocardiographic report, or missing essential demographic data, were excluded from the study.

Echocardiography and diagnostic protocol

Transthoracic echocardiography was performed by experienced pediatric cardiologists, using a standardized protocol in accordance with international guidelines (American Society of Echocardiography, ASE). Examinations were conducted using Philips iE33 (Andover, MA) and GE Vivid S60N (Chicago, IL) ultrasound machines, both equipped with high-resolution pediatric probes. The main cardiac anomalies screened included atrioventricular septal defects (AVSDs), ventricular septal defects (VSDs), atrial septal defects (ASDs), valvular anomalies, and signs of pulmonary arterial hypertension (PAH).

Data collection

Data were collected retrospectively from paper-based medical records archived in the Pediatric Cardiology Unit and the hospital’s electronic medical record system (Hosix, SIVSA, Vigo, Spain). The collected variables included the following: demographic data: age, sex, and geographic origin (urban/rural); referral reason: systematic screening versus symptom-based suspicion; clinical presentation: age at diagnosis and functional signs (dyspnea, cyanosis, and recurrent respiratory infections); echocardiographic findings: type of CHD, the presence of PAH, and other associated cardiovascular anomalies; and management strategy: medical treatment and referral for cardiac surgery.

Missing data

Incomplete records were defined as those lacking a confirmed T21 diagnosis or missing a readable echocardiographic report. No data imputation techniques were used. All such cases were strictly excluded from the final analysis.

Data analysis

Data were entered and analyzed using Microsoft Excel 2016 (Redmond, WA). A descriptive statistical analysis was performed: For quantitative variables, means, medians, and standard deviations were calculated; for qualitative variables, absolute frequencies and percentages were reported. No inferential statistical tests were conducted. However, descriptive subgroup analyses were performed based on age, sex, the type of heart defect, and the presence or absence of PAH.

Ethical considerations

The study received ethical approval from the Ethics Committee of the CHU Mohammed VI in Oujda. Given the retrospective nature of the study, a waiver of informed consent was granted. All data were anonymized in accordance with ethical standards and the principles of the Declaration of Helsinki.

## Results

Between January 2015 and December 2023, a total of 167 children with Down syndrome (trisomy 21, T21) were referred to the pediatric cardiology clinic at Mohammed VI University Hospital Center in Oujda. Among them, 103 were diagnosed with congenital heart defect (CHD) confirmed by echocardiography, representing 61.7% of the total T21 cohort. This corresponds to an annual average of 18.5 cases, with approximately 11 T21 children with CHD diagnosed per year.

The mean age at the first cardiology consultation for T21 children with CHD was 14.4 ± 9.7 months, with a median of 11 months and an interquartile range (IQR) of 5-20 months. Ages ranged from one day to 12 years. The average delay between birth and the cardiologic diagnosis was 13.8 months, indicating a significant delay in diagnosis. Table 1 illustrates our findings.

**Table 1:** The consultation age of children with Down syndrome and confirmed congenital heart defect

The consultation age	Number of cases (n)	Percentage (%)
Birth to one month	9 cases	8.7%
One month to three months	10 cases	9.7%
Three months to six months	12 cases	11.6%
Six months to 12 months	10 cases	9.7%
12 months to 24 months	19 cases	18.4%
24 months to less than five years	28 cases	27.2%
More than five years	15 cases	14.5%
Total	103	100%

The mean maternal age was 38 ± 5.2 years (IQR: 35-42 years), and the mean paternal age was 49 ± 6.9 years (IQR: 45-54 years), with 72.8% of parents being over 40 years old at the time of birth.

Consanguinity was identified in 30.1% of the 103 children with CHD; 21.4% were first degree (first cousins) and 8.7% were second degree (second cousins or more distant).

Regarding the mode and origin of referral, 61.2% (n = 63) were referred due to suggestive clinical signs, 23.3% (n = 24) were referred as part of routine screening following a T21 diagnosis, and 15.5% (n = 16) were referred after the incidental discovery of a heart murmur in the absence of functional symptoms.

Of the symptomatic children (n = 63), 54% came from rural areas, while 34% were referred from neonatal intensive care units (NICUs), 9.5% from general pediatric wards, and 6.5% via outpatient consultations. These findings highlight geographic and structural disparities in access to care.

Among the 61.2% (n = 63) of symptomatic children, several clinical manifestations were noted. The most frequent functional sign was feeding difficulties, reported in 80.9% of the children. This was followed by 68.2% of the children experiencing respiratory distress and 65.1% showing fatigue with exertion. About one-third, or 34.9%, had sweating episodes. A smaller proportion, 14.3%, showed cyanosis. Additionally, 2.9% experienced episodes of fainting (Table 2).

**Table 2:** Functional signs observed in symptomatic children with congenital heart defect

Symptoms	Frequency (%)
Feeding difficulties	80.9%
Respiratory distress	68.2%
Fatigue with exertion	65.1%
Sweating episodes	34.9%
Cyanosis	14.3%
Episodes of fainting	2.9%

A heart murmur was detected during the initial consultation in 60.1% of cases. However, this clinical sign was occasionally overlooked or underestimated by referring facilities, resulting in delayed referral to specialized pediatric cardiology services. Additionally, the physical findings observed in all Down syndrome patients with congenital heart defect are summarized in Table 3.

**Table 3:** Physical signs observed in symptomatic children with congenital heart defect

Physical signs	Number of cases (n)	Percentage (%)
Heart failure associated with a hyperdynamic chest	47 patients	46.5%
Heart murmur	62 patients	60.1%
Low saturation	67 patients	65%

A transthoracic echocardiography was performed in all 103 patients with Down syndrome and congenital heart defect (CHD). This examination enabled an accurate etiological diagnosis, revealing various cardiac anomalies, the distribution of which is presented in Table 4.

**Table 4:** Distribution of congenital heart anomalies in children with Down syndrome (n = 103)

Type		Number of cases	Percentage (%)	Total number of cases (n)	Percentage (%)
Atrioventricular septal defects (AVSDs)	AVSD complete with pulmonary hypertension (PAH)	25 patients	24.3%	40 patients	38.8%
	AVSD complete without PAH	10 patients	9.7%		
	Partial AVSD	5 patients	4.8%		
Ventricular septal defects (VSDs)	Minimal VSD	7 patients	6.8%	32 patients	31%
	Large VSD	9 patients	8.7%		
	Large VSD with PAH	16 patients	15.5%		
Atrial septal defects (ASDs)	ASD with PAH	1 patient	0.9%	22 patients	21.3%
	Minimal ASD	13 patients	12.6%		
	Large ASD	8 patients	7.8%		
Patent ductus arteriosus (PDA)				3 patients	2.9%
Tetralogy of Fallot				3 patients	2.9%
Transposition of the great arteries				1 patient	0.9%
Pulmonary stenosis				1 patient	0.9%
Hypoplasia of the aortic isthmus				1 patient	0.9%
Total				103 patients	100%

**Figure 1:**
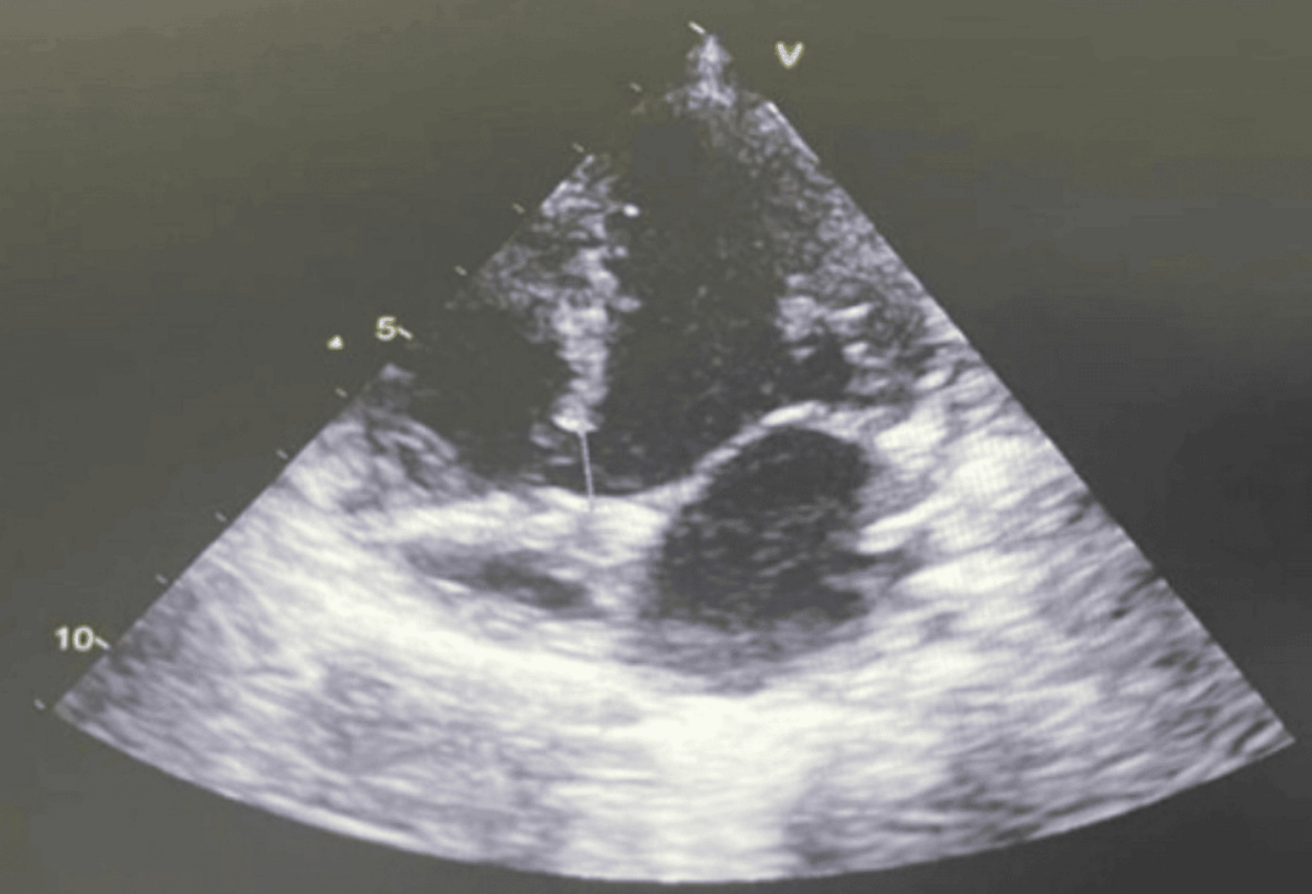
Four-chamber view showing a ventricular septal defect (VSD)

**Figure 2:**
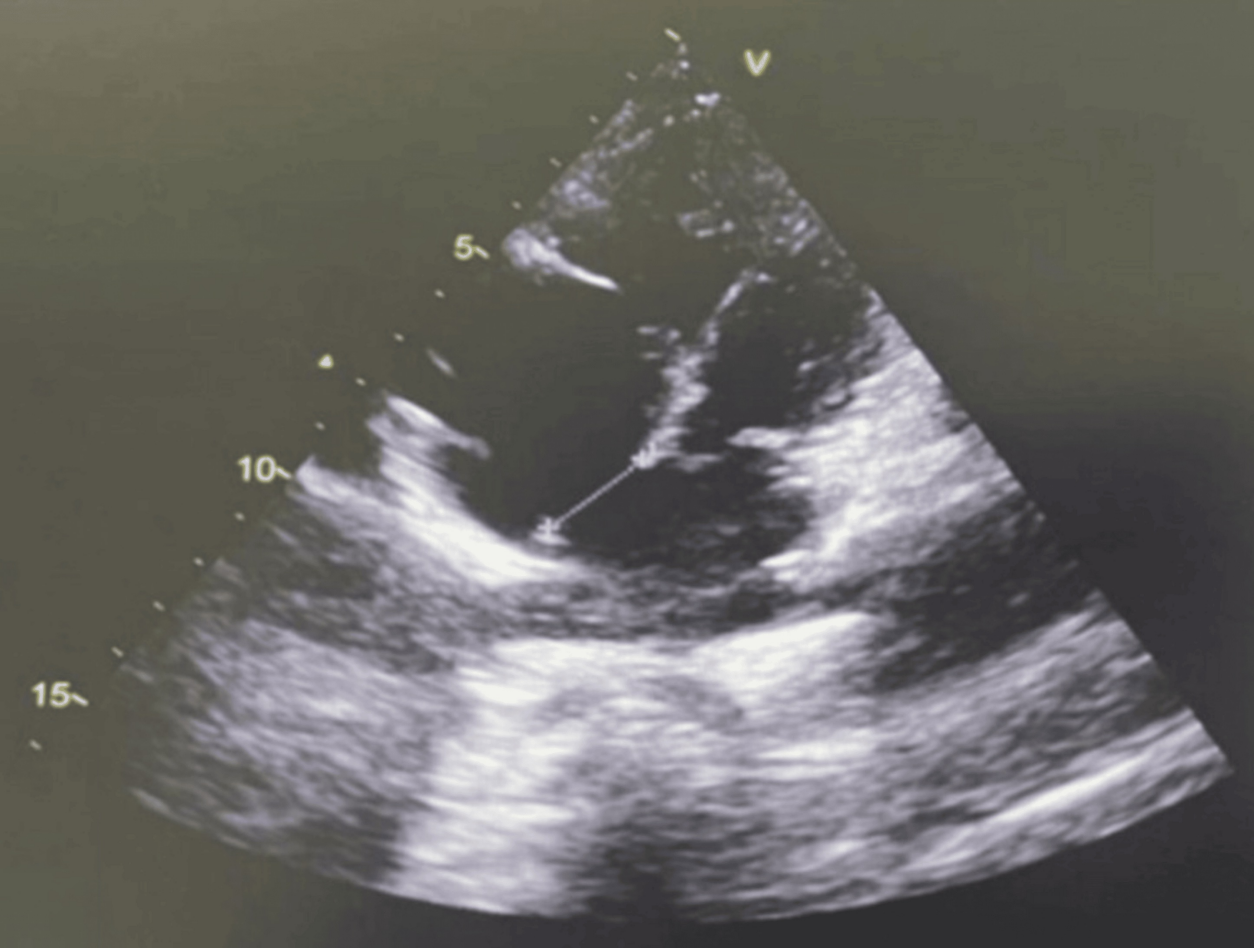
Four-chamber view showing an atrial septal defect (ASD)

Atrioventricular septal defects (AVSDs) were the most frequent malformations, observed in 40 patients (38.8% of the total cohort, n = 103). Ventricular septal defects (VSDs) were the second most common (Figure 1), found in 32 patients (31.1%), followed by atrial septal defects (ASDs) in 22 (21.3%) children (Figure 2). Among these septal anomalies, size was considered for therapeutic indications; VSDs were classified as large (>5 mm), moderate (3-5 mm), or small (<3 mm). ASDs were considered significant if their diameter exceeded 8 mm or if they were associated with a significant left-to-right shunt and chamber dilatation.

Other congenital anomalies included patent ductus arteriosus (PDA) (2.9%), tetralogy of Fallot (ToF) (2.9%), transposition of the great vessels (TGV) (0.9%), pulmonary stenosis (0.9%), and hypoplasia of the aortic isthmus (0.9%).

Among the 103 children, 39.8% (n = 41) presented with pulmonary arterial hypertension (PAH). It was already present at the first consultation in 71% of cases, indicating a significant diagnostic delay, and developed during follow-up in the remaining 29%. PAH diagnosis was based on the echocardiographic estimation of systolic pulmonary artery pressure using tricuspid regurgitation velocity, with a threshold value of >35 mmHg, along with right heart chamber dilatation or paradoxical septal motion. No cardiac catheterization was performed.

Regarding surgical management, 62 children (60.2%) had an indication for surgery, as defined by international guidelines (shunt size, chamber overload, and clinical impact). However, only 14 patients (22.6% of those eligible) actually underwent cardiac surgery. The main reasons for not undergoing surgery were irreversible pulmonary arterial hypertension (PAH) (n = 28 or 45.2% of non-operated cases), parental refusal or loss to follow-up (n = 20), and financial or logistical constraints (n = 12).

Among the 40 cases of AVSD, only four patients without PAH (17.4%) underwent surgery. The remaining children were not operated on due to advanced PAH or parental refusal. Of the 32 VSD cases, nine closed spontaneously (28.1%). Among the 19 children with a surgical indication, six underwent surgery (31.6%). Of the 22 ASDs, 10 minor forms (45.4%) evolved toward spontaneous closure. The significant forms remain untreated to date. Surgery was performed in two out of three PDA cases (66.6%). Complex anomalies (ToF, TGV, pulmonary stenosis, and isthmic hypoplasia) were managed individually, depending on access to a specialized center and the clinical condition at the time of diagnosis (Table 5).

**Table 5:** Surgical management of congenital heart defects in children with trisomy 21 (n = 103) AVSD, atrioventricular septal defect; VSD, ventricular septal defect; ASD, atrial septal defect; PDA, patent ductus arteriosus; ToF, tetralogy of Fallot; TGV, transposition of the great vessels; PAH, pulmonary arterial hypertension

Type of malformation	Total (n)	Surgical indication (n)	Operated patients (n)	Parental refusal/loss to follow-up (n)	Pulmonary hypertension contraindicating surgery (n)	Spontaneous closure (n)
AVSD	40	23 (sans PAH)	4	8	17	-
VSD	32	19	6	5	8	9
ASD	22	12 (significant forms)	0	6	-	10
PDA	3	3	2	0	1	0
ToF/TGV/others	6	5	2	1	2	0
Total	103	62	14	20	28	19

These findings highlight not only the high frequency of severe congenital heart defects in children with trisomy 21 but also the numerous barriers to optimal management, particularly when delayed diagnosis leads to irreversible pulmonary hypertension. The low proportion of children undergoing surgery despite clear indications reflects significant disparities in access to specialized care in our regional context. These observations reinforce the need to strengthen systematic early screening for congenital heart defects in newborns with trisomy 21, especially in rural or peripheral areas.

## Discussion

Trisomy 21 is often described as a “chromosomal anomaly” or “chromosomal aberration,” as reported in several sources addressing Down syndrome [2,3]. This condition is primarily characterized by the presence of an extra chromosome 21, specifically in the 21q22 region, where the genes responsible for the disease phenotype are located.

While John Langdon Down initially described circulation as “weak” in his original description of the syndrome in 1866, it was Alfred Garrod in 1894 who specifically noted an association between this syndrome and cardiac malformations, marking a significant advancement in understanding the cardiologic aspects of trisomy 21 [4,5].

The prevalence of congenital heart defects among individuals with trisomy 21 is remarkably high. Approximately 40%-70% of individuals with trisomy 21 have a congenital heart defect [6]. In our study, 62.4% of the 165 children with trisomy 21 had a congenital heart defect. In England, 47.8% of the 142 cases studied had a heart malformation [7]. Finally, in Senegal, 40.5% of the 257 individuals with trisomy 21 had congenital heart defect [8].

Thus, antenatal diagnosis has become a cornerstone in the management of congenital heart defects, with a detection rate, for all heart defects combined, of 8% in 1983-1987 and 60% by 2000 [9,10].

The facial features of infants with trisomy 21 often provide early clinical clues to the presence of this chromosomal anomaly, which is frequently associated with congenital heart defects such as ventricular septal defects (VSDs), atrioventricular canal defects (AVCD), patent ductus arteriosus (PDA), and, occasionally, tetralogy of Fallot [11]. Typical craniofacial characteristics include upward-slanting palpebral fissures, a flat facial profile with a depressed nasal bridge, epicanthal folds, small low-set ears, and a protruding tongue due to relative macroglossia and hypotonia. Additional features such as a short, broad neck with excess nuchal skin and downward-turned corners of the mouth may further support the clinical diagnosis. The recognition of these features should prompt early genetic testing and cardiovascular evaluation.

The distribution of children with trisomy 21 and congenital heart defect based on age at diagnosis reveals interesting trends that illustrate a certain evolution in screening practices over time.

International studies show notable variations in the age at diagnosis of congenital heart defect in children with trisomy 21. In the United States, a study revealed that a significant proportion (37%) of cases were detected between the ages of six and 10 years [12]. This distribution suggests a trend toward later diagnosis, with a substantial concentration of cases in the 6-10-year age group. In Algeria, a study showed that 7% of children were diagnosed from birth to one month, 21% between one and three months, and 14% between three and six months [13]. A notable proportion of 43% of diagnoses were made after one year, indicating a trend toward later diagnosis.

In comparison, our study shows that the most represented age group is from 24 months to less than five years, with 27.2% of cases diagnosed within this period. The average age at diagnosis was 14 months and 11 days, with a range from one day to 12 years.

The data from our study reveal a persistent challenge in the early diagnosis of congenital heart defects in children with Down syndrome. This delay can lead to severe complications such as Eisenmenger syndrome, which complicates medical management and negatively affects the prognosis of the patients.

Advanced parental age is a well-documented risk factor for chromosomal abnormalities, including Down syndrome, and is also associated with an increased risk of congenital heart defects. Medical literature highlights that this risk is primarily related to advanced maternal age, but paternal age also plays a significant role. In our study, there was a predominance of parents aged over 40 years.

Consanguinity is a well-documented risk factor for genetic abnormalities, including Down syndrome and its complications such as congenital heart defects.

Apart from patients referred for prenatal screening or systematic cardiac screening due to Down syndrome, several indicative signs can suggest the presence of heart defects in these patients: poor weight gain, cyanosis in infants, feeding difficulties, psychomotor developmental delay, sweating during feedings (bottles), respiratory distress, fainting and syncope, and finally palpitations described by older children [14].

In terms of symptoms, the results of our study reveal that the most frequently observed manifestations were feeding difficulties (80.9%), respiratory discomfort (68.2%), and fatigue during exertion (65.1%). In comparison, a study conducted in Senegal [8] found that 39.6% of children with Down syndrome and congenital heart defect presented with dyspnea, while a study in Algeria [13] observed cyanosis in 53.8% of cases.

Regarding heart murmurs, our study showed that they were present in 60.2% of children, a slightly lower rate than that observed in 90.5% in Senegal [8]. As for cyanosis, it was observed in 14.3% of cases in our study, compared to 11.5% in Senegal [8] and 53.8% in Algeria [13], indicating variations in the frequency of this symptom. For heart failure, 4.8% of children in our study presented signs, compared to 9.4% in Senegal [8] and 38.5% in Algeria [13], reflecting differences in the prevalence of this symptom. Finally, our study revealed that 5.8% of children had low oxygen saturation and 9.7% exhibited hyperdynamic thoraxes, highlighting the importance of a comprehensive clinical evaluation to detect signs of congenital heart defect in children with Down syndrome.

The variations observed in the frequency of heart murmurs, cyanosis, and heart failure across different studies may be explained by several factors, including differences in the age of children at the time of evaluation, the nature and distribution of congenital heart defects, and the clinical assessment methods used. For example, certain symptoms may not be apparent in the early stages of life or may vary depending on the type of defect. In addition, disparities in access to healthcare and delays in diagnosis or referral can lead to more advanced presentations in some settings. Differences in sample size and selection criteria may also influence the reported prevalence. Standardizing clinical evaluation methods and implementing systematic neonatal screening for congenital heart defect would facilitate more accurate comparisons between countries and healthcare systems.

International studies consistently show that the atrioventricular canal defect (AVCD) is the most common congenital heart defect in children with Down syndrome, although the prevalence of other defects can vary significantly across different regions. This underscores the global differences in the types of heart malformations observed in children with Down syndrome.

In the United States [12], AVCD is found in 40% of cases, while in Algeria [13], the prevalence is 21%. In our study, AVCD represents 38.8% of the cases, aligning closely with the US figure.

The ventricular septal defect (VSD) is also a frequent heart malformation. In the United States, VSD occurs in 30% of cases, which is similar to our study, where it accounts for 31% of cases [12]. In Algeria, the prevalence of VSD is 29% [13].

Atrial septal defects (ASDs) exhibit considerable variation in prevalence across studies. In Algeria, 21% of patients have ASD [13]. In the United Kingdom [7], the prevalence is 17.7%, while in the United States [12], it is lower, at just 10%. In our study, ASD is observed in 21.3% of cases.

The prevalence of patent ductus arteriosus (PDA) varies as well. In Algeria [13], PDA is found in 21% of patients, while in the United States [12], it is present in 16%. In our study, however, the prevalence of PDA is relatively low, at 2.9%.

As echocardiograms were only conducted on symptomatic patients, the actual incidence of patent ductus arteriosus (PDA) in our study may be underreported.

Upon birth, prompt management by a neonatology team is crucial for the continuous monitoring of the cardiac and respiratory functions of newborns with Down syndrome and congenital heart defects.

Recent advancements in the medical management of congenital heart defects (CHDs) include the use of prostaglandins, angiotensin-converting enzyme inhibitors, and selective pulmonary vasodilators, such as inhaled nitric oxide and endothelin receptor antagonists. These innovations have significantly enhanced postoperative care for high-risk procedures and provide new treatment options for pulmonary hypertension, whether primary or secondary [15,16].

Interventional catheterization, on the other hand, represents an effective therapeutic alternative, either palliative or curative, that can be performed at any age. Many congenital heart defects can be managed without the need for open-heart surgery [17,18].

Depending on the type of congenital heart defect diagnosed, surgical intervention may be considered in the first days of life or during the first year, aiming to optimize clinical outcomes and improve the quality of life for these particularly vulnerable children [9,19].

The current goal of such surgery is not only to alter the immediate life prognosis but also, and more importantly, to interrupt as early as possible the harmful pathophysiological consequences of these conditions, as they are optimally correctable [18].

In children with Down syndrome, particularly those with a complete atrioventricular canal (CAV), the progression to Eisenmenger syndrome can occur more rapidly if the congenital heart defect is not addressed early. This condition is typically a result of severe, irreversible pulmonary hypertension.

Thanks to surgical interventions and medical treatments, patients with Down syndrome and congenital heart defects now experience a significantly improved life expectancy. Atrioventricular communication is the most prevalent congenital heart defect in these patients and is associated with a high mortality rate. Around 33% of Down syndrome patients with CAV develop pulmonary arterial hypertension, with 25% of these patients dying during follow-up [20].

Unfortunately, long-term follow-up data were not available for our cohort. This is a clear limitation, as it prevents us from evaluating surgical outcomes, complications, and the quality of life of these children over time. Additionally, the low rate of surgical intervention observed in our study raises important questions. Some children may have been ineligible for surgery due to irreversible pulmonary hypertension, while others may have faced systemic barriers such as limited surgical slots, geographic or financial constraints, or parental refusal. The unavailability of advanced medical therapies in some settings, such as inhaled nitric oxide, endothelin receptor antagonists, or prostacyclin analogs, further limits treatment options, especially for those with advanced disease.

These findings highlight an urgent need to improve early detection and care pathways for children with Down syndrome and CHD. Our data support the implementation of standardized local or national screening protocols, such as mandatory echocardiographic evaluation of all newborns with trisomy 21, ideally within the first few weeks of life. Early diagnosis would enable better planning for intervention, reduce preventable complications, and improve overall outcomes. In addition, the development of structured referral pathways and regional networks could help optimize access to pediatric cardiology and cardiac surgery services.

Limitations of the study

This study has several limitations that should be acknowledged.

Retrospective Design

The retrospective nature of the study, based on preexisting medical records, may have introduced inconsistencies in data collection. Variability in clinical documentation and the absence of standardized echocardiographic reporting protocols could affect the reliability and completeness of the information analyzed. Additionally, potential interobserver variability in echocardiographic interpretation must be considered, especially in the absence of uniform diagnostic criteria.

Single-Center Study

Conducted at a single tertiary care hospital (CHU Mohammed VI, Oujda), the findings may not be fully generalizable to other healthcare institutions or regions of Morocco, particularly in rural or underserved settings where access to pediatric cardiology services is more limited.

Limited Sample Size

Although 167 children with trisomy 21 were included, only 103 had confirmed congenital heart defects (CHDs). This relatively small sample size may reduce statistical power and limit the detection of less common cardiac anomalies or subtle associations between variables.

Potential Selection Bias

Only patients referred for pediatric cardiology consultation and documented in the hospital database were included in the analysis. This may have excluded asymptomatic children or those with mild, undiagnosed defects such as small atrial septal defects (ASDs) or patent ductus arteriosus (PDA), leading to the underestimation of the true prevalence of CHDs in this population.

Incomplete Clinical and Diagnostic Data

Missing data for key variables, such as age at diagnosis, presenting symptoms, and detailed echocardiographic findings, were noted. Despite efforts to retrieve missing information from medical files, this limitation may have introduced bias and restricted the depth of statistical analysis.

Evolving Diagnostic Practices

Over the study period, institutional protocols and access to diagnostic technologies (e.g., echocardiography quality and operator expertise) may have evolved, possibly affecting the consistency of case detection. Changes in clinical practices could influence the observed prevalence and types of CHDs diagnosed.

Lack of Long-Term Follow-Up Data

The study focused primarily on initial diagnosis and early management. Data on long-term outcomes, surgical interventions, complications, and patient quality of life were not available, limiting the ability to assess prognosis and the effectiveness of treatment strategies.

## Conclusions

The management of children with Down syndrome and congenital heart defects (CHDs) in Morocco remains suboptimal due to multiple interrelated factors. These include the absence of systematic prenatal screening for aneuploidies, legal restrictions on the medical termination of pregnancy even in confirmed cases, and limited access to specialized care. Such constraints, influenced by legal, ethical, and cultural considerations, restrict early diagnosis and intervention opportunities, ultimately affecting the care pathways and outcomes of affected children.

To improve care, a coordinated multidisciplinary model is essential, involving prenatal and postnatal collaboration between obstetricians, neonatologists, pediatric cardiologists, and other specialists. Key interventions should include early echocardiographic screening, centralized referral systems, and increased awareness among families and healthcare providers. Furthermore, prospective multicenter studies with long-term follow-up are urgently needed to inform national health strategies and ensure better outcomes for this vulnerable population.
